# A model of the aged lung epithelium in idiopathic pulmonary fibrosis

**DOI:** 10.18632/aging.203291

**Published:** 2021-07-08

**Authors:** Hoora Shaghaghi, Karina Cuevas-Mora, Rachel Para, Cara Tran, Willy Roque, Matthew J. Robertson, Ivan O. Rosas, Ross Summer, Freddy Romero

**Affiliations:** 1 Department of Medicine, Division of Pulmonary, Allergy and Critical Care and the Center for Translational Medicine, The Jane and Leonard Korman Respiratory Institute, Philadelphia, PA 19107, USA; 2 Department of Medicine, Rutgers – New Jersey Medical School, Newark, NJ 07103, USA; 3 Pulmonary, Critical Care and Sleep Medicine, Baylor College of Medicine, Houston, TX 77030, USA

**Keywords:** aging, IPF, mitochondria, proteostasis, epithelial cells

## Abstract

Idiopathic pulmonary fibrosis (IPF) is an age-related disorder that carries a universally poor prognosis and is thought to arise from repetitive micro injuries to the alveolar epithelium. To date, a major factor limiting our understanding of IPF is a deficiency of disease models, particularly *in vitro* models that can recapitulate the full complement of molecular attributes in the human condition. In this study, we aimed to develop a model that more closely resembles the aberrant IPF lung epithelium. By exposing mouse alveolar epithelial cells to repeated, low doses of bleomycin, instead of usual one-time exposures, we uncovered changes strikingly similar to those in the IPF lung epithelium. This included the acquisition of multiple phenotypic and functional characteristics of senescent cells and the adoption of previously described changes in mitochondrial homeostasis, including alterations in redox balance, energy production and activity of the mitochondrial unfolded protein response. We also uncovered dramatic changes in cellular metabolism and detected a profound loss of proteostasis, as characterized by the accumulation of cytoplasmic protein aggregates, dysregulated expression of chaperone proteins and decreased activity of the ubiquitin proteasome system. In summary, we describe an *in vitro* model that closely resembles the aberrant lung epithelium in IPF. We propose that this simple yet powerful tool could help uncover new biological mechanisms and assist in developing new pharmacological tools to treat the disease.

## INTRODUCTION

Idiopathic pulmonary fibrosis (IPF) represents one of the most aggressive and irreversible lung diseases, has an unknown etiology, and limited therapeutic options [1]. The existing paradigm is that IPF arises from multiple, low-grade insults to the alveolar epithelium, which exhaust critical stem cell capacity and activate pathological signaling pathways that result in progressive tissue remodeling [2–5].

Multiple risk factors have been linked to the pathogenesis of IPF, including but not limited to genetic mutations and environmental exposures [6–8]. However, age represents the most significant risk factor for the development of IPF [3, 5, 9, 10]. Indeed, IPF is a condition that rarely develops before the age of 50, and shows a nearly exponential rise in incidence after the seventh decade of life. Consistent with this association, multiple cell types within the IPF lung have been shown to manifest cellular and molecular hallmarks of pathologically aging tissues [9, 11].

A defining feature of the aged IPF lung is that alveolar epithelial type II (AE2) cells lose the capacity to proliferate and adopt features of senescent cells [9, 12–14]. Senescent cells are also believed to drive immune cell and fibroblast activation via their production of senescence-associated secretory proteins (SASP), such as IL-6, TNFα- and TGF-β. Consistent with this paradigm, emerging evidence indicates that experimentally-induced pulmonary fibrosis can be ameliorated by targeting senescent cells for destruction [15], suggesting a role for senolytic therapies in the treatment of IPF. In addition to cellular senescence, other notable hallmarks of aging linked to IPF include telomere attrition, mitochondrial dysfunction, altered cellular metabolism and loss of proteostasis, all of which are considered potential areas of therapeutic intervention.

Although abnormal aging is believed to underlie the pathogenesis of IPF, surprisingly little is known of the mechanisms driving pathologically aging in this disease. With this in mind, our goal was to develop a better *in vitro* model; one that more closely resembles the features of aged IPF lung tissues. Our central hypothesis was that existing *in vitro* models do not recapitulate major features of aging IPF tissues, in large part, because they rely on one-time exposures, rather than repetitive insults seen by lungs of IPF patients. In this study, we describe a novel 7-day injury protocol that reliably induces phenotypic and functional characteristics of IPF in cultured mouse alveolar epithelial cells. Based on these findings, we hypothesize that our model system may help to uncover new biological mechanisms and assist in identifying novel pharmacological agents that can be used to target the alveolar epithelium.

## RESULTS

### A seven-day injury protocol that induces cellular senescence in MLE12 cells

**Figure 1:**
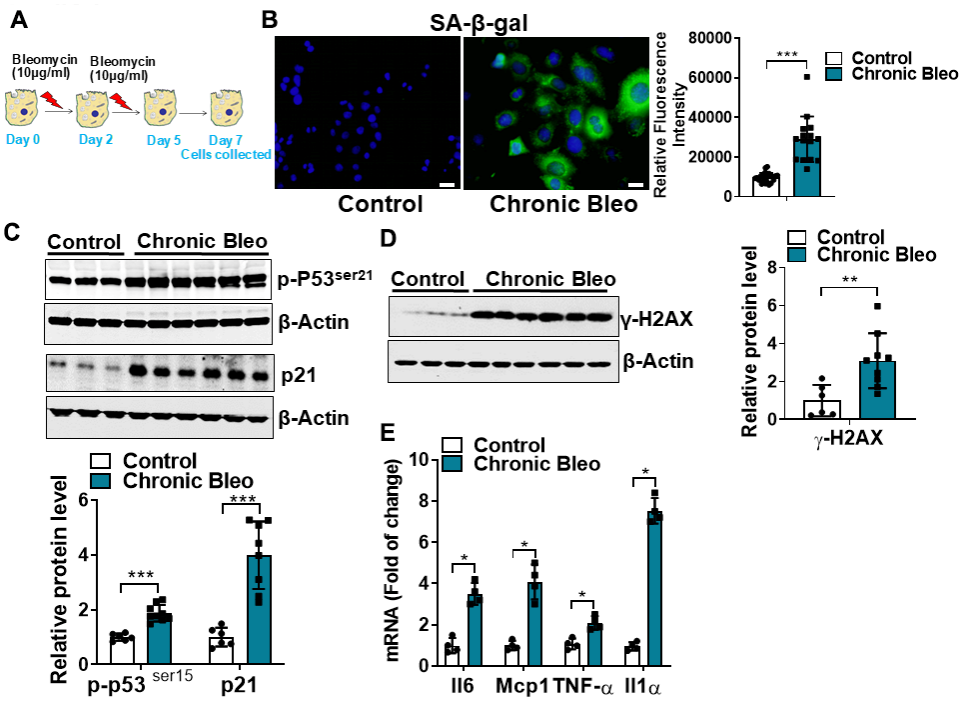
**Cellular senescence markers are increased in MLE12 cells after our 7-day injury protocol.** (**A**) Schematic showing the experimental design: On day 0, MLE-12 cells were treated with 10 μg/ml bleomycin for 24h. Day 2, bleomycin was removed, and the culture medium was refreshed. Day 4, cells were treated again with 10 μg/ml bleomycin. Day 5, bleomycin was subsequently removed, and cells were collected at day 7. (**B**) SA-β-gal activity (green fluorescence cytoplasmic staining) in control and chronically injured MLE12 cells. Number of SA-β-gal positive cells per 100 cells counted (right). (**C**) Western blot (WB) for p-p53*^Ser21^* and p21 in chronically injured lung epithelial cells (with β-actin loading control). Densitometry is shown on the right. (**D**) WB for γ-H2AX in control and bleomycin injured cells (with β-actin loading control). Densitometry is shown on the right. (**E**) Transcript levels for Il6, Mcp1, Tnf-α, and Il1α in controls vs. chronically injured lung epithelial cells. Statistical significance was assessed by unpaired Student’s t-test for two groups. * p< 0.05, **P < 0.01, ***P < 0.001 versus control group, n=6.

Cellular senescence is key characteristic of the alveolar epithelium in IPF. With this in mind, we first sought to develop an injury protocol that could reliably induce this pathological feature [12, 16–18]. After screening various concentrations [10, 25, 50 μg/ml), durations (24 or 48 hr), and frequencies (every 48 or 72hr) of bleomycin in MLE12 cells, we uncovered a protocol (Figure 1A) that reliably induced features of senescent cells. This included causing the characteristic flattening and enlarging of cell shapes as well as the suppression of cell proliferation (data not shown). We also detected an upregulation in β-galactosidase activity (Figure 1B), and higher expression of multiple cellular senescence markers, including the cell cycle inhibitors p21 and p53, the DNA damage marker γ-H2AX and the SASPs IL-6, MCP1, TNF-α, and IL-1α (Figure 1C–1E). Altogether, these findings indicate that our unique 7-day injury protocol induces multiple characteristic features of senescent IPF cells.

### Mitochondrial function is impaired in MLE12 cells undergoing our seven-day injury protocol

**Figure 2:**
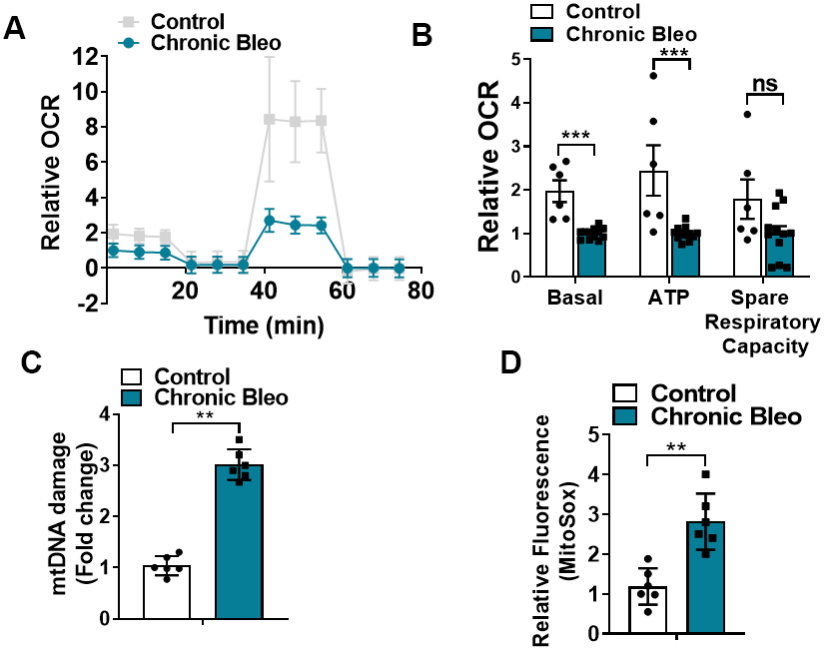
**7-day bleomycin protocol disrupts mitochondrial homeostasis in alveolar epithelial cells.** (**A**, **B**) Mitochondrial oxygen consumption in control and chronically injured MLE12 cells. Right, bar graph depicting basal oxygen consumption rate (OCR), ATP production and spare respiratory capacity. (**C**) mtDNA damage quantification in control and bleomycin injured cells. (**D**) Quantification of reactive oxygen species (ROS) by MitoSox staining in control and chronically injured MLE12 cells. Seahorse data are representative of 3 separate experiments. Statistical significance was assessed by unpaired Student’s t-test except for Seahorse analyses in which two-way analysis of variance (ANOVA) followed by Sidak post hoc analysis was used to adjust for multiple comparisons. Statistical significance was achieved when P < 0.05 at 95% confidence interval. Given multiple * p< 0.05, **P < 0.01, ***P < 0.001 versus control group, n=6.

**Figure 3:**
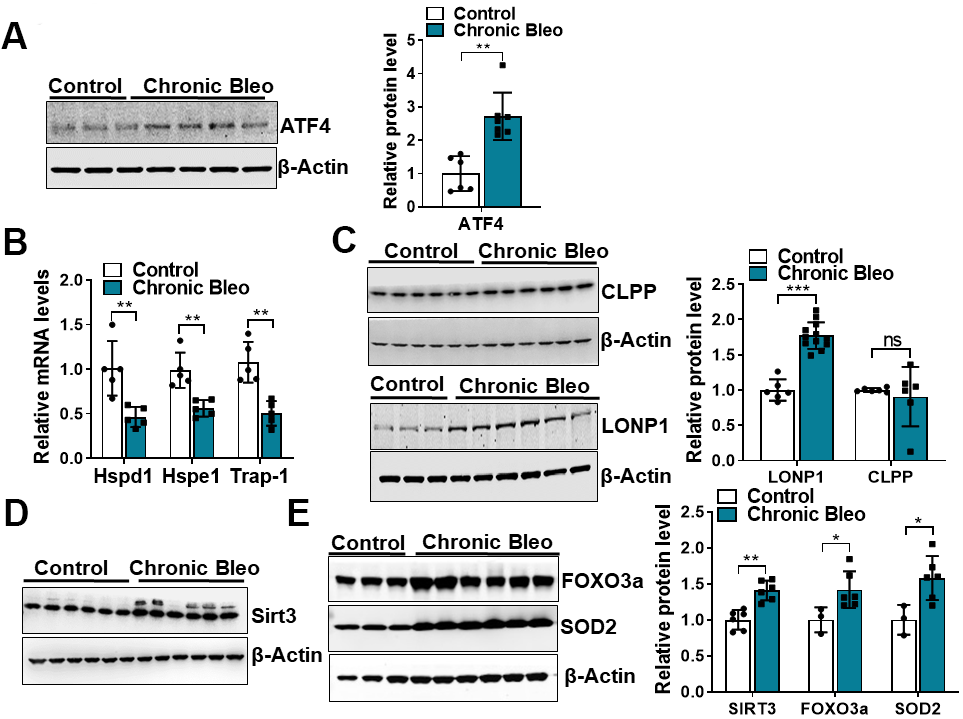
**7-day bleomycin protocol activates the mitochondria unfolded protein response and induces expression of mitochondrial chaperones in alveolar epithelial cells.** (**A**) WB for ATF4 in controls vs. chronically injured lung epithelial cells (with β-actin loading control). Densitometry is shown on the right. (**B**) Transcript levels for Hsp60, Hsp10 and Hps90 in controls vs. chronically injured MLE12 cells. (**C**) WB for Clpp and Lonp1 in control and bleomycin injured cells (with β-actin loading control). Densitometry is shown on the right. (**D**, **E**) WB for SIRT3, FOXO3a and SOD2 in control and bleomycin injured MLE12 cells (with β-actin loading control). Densitometry is shown on the right. Statistical significance was assessed by unpaired Student’s t-test for two groups. * p< 0.05, **P < 0.01, ***P < 0.001 versus control group, n=6.

Like cellular senescence, mitochondrial dysfunction has emerged as a key feature of the alveolar epithelium in IPF. To determine whether mitochondrial dysfunction was altered in our model system, we began by comparing mitochondrial oxygen consumption rates (OCR) between control and injured cells. Consistent with altered mitochondrial function, we found that OCR was significantly reduced in cells undergoing our injury protocol. This included a nearly 50% reduction in basal respiration as well as an even greater decline in ATP production (Figure 2A, 2B). Further, we found that DNA damage and redox balance (Figure 2C, 2D) were altered in these cells and we detected higher expression of ATF4, a transcriptional regulator of the mitochondrial unfolded protein response (UPR^mt^) (Figure 3A). Consistent with the upregulation in ATF4, we observed an increase in several of its downstream targets, including the mitochondrial chaperone proteins Hspd1, Hspe1 and Trap1 and mitochondrial proteases CLPP and Lonp1 (Figure 3B, 3C). Lastly, we detected mitochondrial changes not yet described in the alveolar epithelium of IPF patients, such as an increase in levels of the deacetylase SIRT3, the transcription factor forkhead box O3a (FOXO3) and the antioxidant enzyme superoxide dismutase 2 (SOD2) (Figure 3D, 3E), suggesting our model can help to uncover new mechanisms in the pathogenesis of disease.

### Cellular metabolism is altered in our seven-day bleomycin injury model

**Figure 4:**
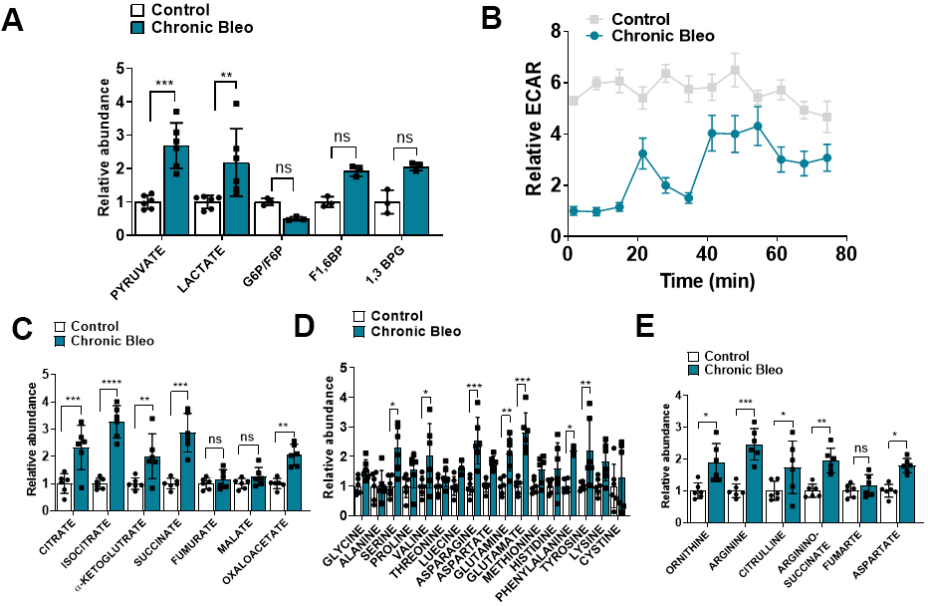
**7-day bleomycin protocol alters cellular metabolism in alveolar epithelial cells.** (**A**) Levels of glycolytic metabolites in control and bleomycin injured cells. (**B**) Extracellular acidification rate in control and bleomycin injured cells. (**C**–**E**) Tricarboxylic acid (TCA) cycle intermediates, amino acid levels and urea cycle intermediates in control and bleomycin injured cells. Statistical comparisons were performed using two-way analysis of variance (ANOVA) followed by Sidak post hoc analysis in order to adjust for multiple comparisons. * p< 0.05, **P < 0.01, ***P < 0.001 versus control group, n=6.

Another recently described feature of IPF cells is dysregulated cellular metabolism [19–22]. To determine whether cellular metabolism was affected in our model, beyond the changes already described in OCR, we measured levels of different metabolites by LC/MS in control and injured cells. Consistent with findings described in IPF, we uncovered significantly higher levels of lactate in injured cells [23]. This also associated with elevated pyruvate levels and a trend toward higher levels of fructose 1,6-biphosphate (F1,6BP) and 1, 3-bisphosphate glycerate (1,3 BPG) (Figure 4A), suggesting glycolysis was upregulated in these cells. However, this did not correlate with higher extracellular acidification rates (levels were actually significantly lower), suggesting, at the very least, that lactate secretion was reduced in injured cells (Figure 4B). In addition to elevated levels of glycolytic intermediates, we also detected higher levels of several TCA cycle intermediates (citrate, isocitrate, α-ketoglutarate and succinate) (Figure 4B) and various amino acids, including the nitrogen-rich amino acids glutamine, asparagine and arginine (Figure 4C) [24, 25]. Consistent with altered nitrogen balance, multiple metabolites of the urea cycle were increased in our bleomycin-injured MLE12 cells (Figure 4E).

### Our seven-day injury model induces a loss of proteostasis in MLE12 cells

**Figure 5:**
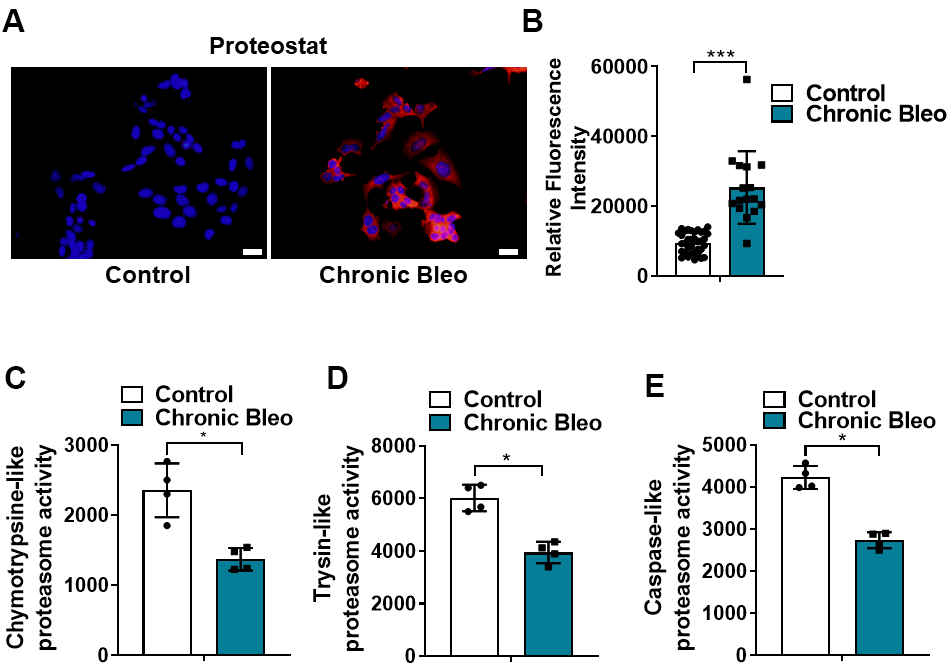
**7-day bleomycin protocol leads to loss of proteostasis in alveolar epithelial cells.** (**A**, **B**) Proteostat staining (aggresome) in controls and chronically injured MLE12 cells. Quantification is shown on the right. (**C**–**E**) Chymotrypsin-like, trypsin-like and caspase-like activities in control and bleomycin injured MLE12 cells. Statistical significance was assessed by Student t-test **P < 0.01, ***P < 0.001 versus control group, n=6.

**Figure 6:**
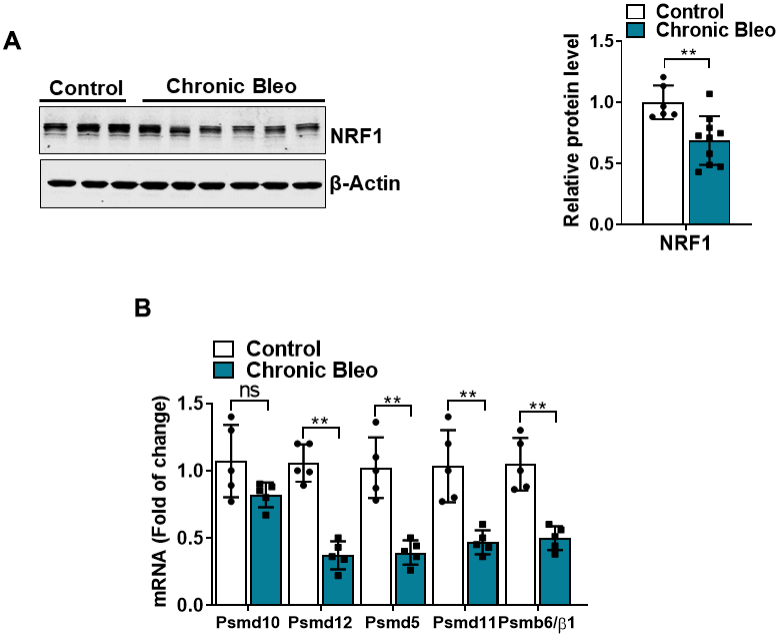
**7-day bleomycin protocol reduces NRF-1 expression in alveolar epithelial cells.** (**A**) WB for NRF1 in control and bleomycin injured cells (with β-actin loading control). Densitometry is shown on the right. (**B**) Transcript levels for PSMD10, PSMD12, PSMD5, PSMD11, and PSMB6/β1 in controls vs. injured MLE12 cells. Statistical significance was assessed by unpaired Student’s t-test for two groups. * p< 0.05, **P < 0.01, ***P < 0.001 versus control group, n=6.

Another defining feature of IPF cells is a loss of proteostasis. For example, mutations causing the misfolding of surfactant proteins are linked to familial forms of IPF and misfolded and aggregated proteins have been shown to accumulate in the alveolar epithelium of patients with sporadic forms of disease [26, 27]. To assess whether proteostasis was altered in our model system, we first assessed levels of intracellular protein aggregates in injured vs uninjured cells. Consistent with impaired proteostasis, we found that intracellular protein aggregates were significantly increased in injured cells (Figure 5A, 5B). Further, this associated with homeostatic changes in the ubiquitin proteasome system (UPS), including marked reduction in chymotrypsin-, trypsin-, and caspase-like activities (Figure 5C, 5E) as well as significant accumulation of K48-linked ubiquitinated proteins (data not shown). Further, we detected reduced levels of various UPS proteins, such as the transcriptional regulator Nuclear E2-related factor 1 (NRF1) (Figure 6A), and several key components of the 26S proteosome (Psmd12, Psmd5 and PsmbB6/β1) (Figure 6B).

**Figure 7:**
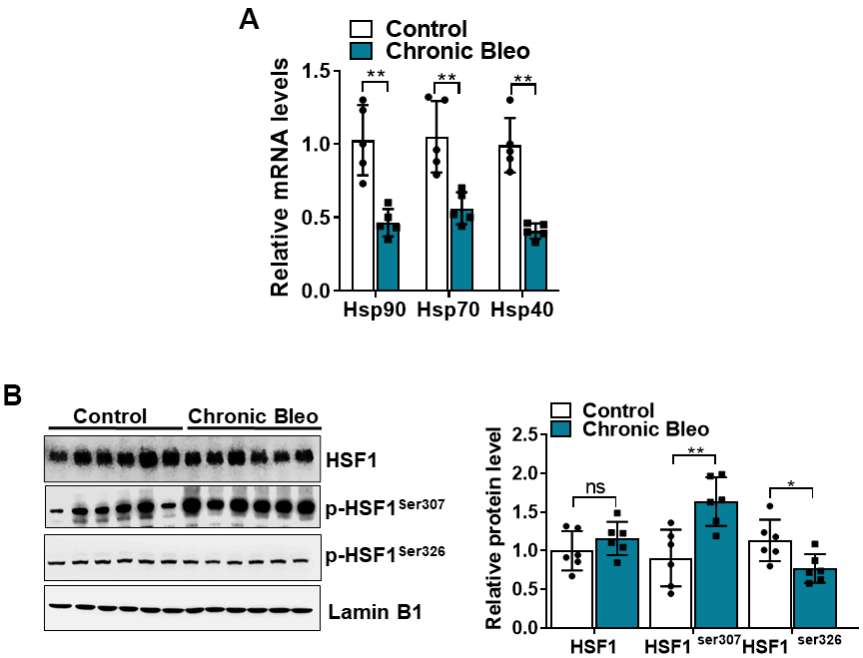
**The HSF1 activity is impaired in MLE12 cells after our 7-day injury protocol.** (**A**) Transcript levels for Hsp90, Hsp70, and Hsp40 in control and bleomycin injured MLE12 cells. (**B**) WB for HSF1, p-HSF1^ser307^, p-HSF1^ser326^ in control, and bleomycin injured MLE12 cells (with Laminin-B1 loading control). Densitometry is shown on the right. Results of densitometry analysis are depicted in bar graphs (n= 6, per group). Statistical significance was assessed by unpaired Student’s t-test for two groups. * p< 0.05, **P < 0.01, ***P < 0.001 versus control group, n=6.

Having established that our model impairs proteostasis, we next sought to uncover other mechanisms that contribute to loss of proteostasis. Since chaperone proteins are critically involved in the trafficking and folding of proteins, we next sought to determine whether expression of these proteins was altered in injured cells. Consistent with the loss of proteostasis, we found that transcript levels for heat shock proteins (HSP)-*70, -90,* and *-40* were significantly reduced in injured versus uninjured cells (Figure 7A). Further, this associated with various protein modification of heat shock transcription factor 1 (HSF1) (Figure 7B), which is a key regulator of many HSP genes. Although total protein levels were not significantly different, we detected a nearly 1.5 fold increase in levels of the inactivating modification at *Ser307* (Figure 7B), and a 30% reduction in levels of the activating phosphorylation at S*er326*. Taken together, these findings support the notion that our model could help to advance our understanding of why IPF causes a loss of proteostasis.

## DISCUSSION

**Figure 8:**
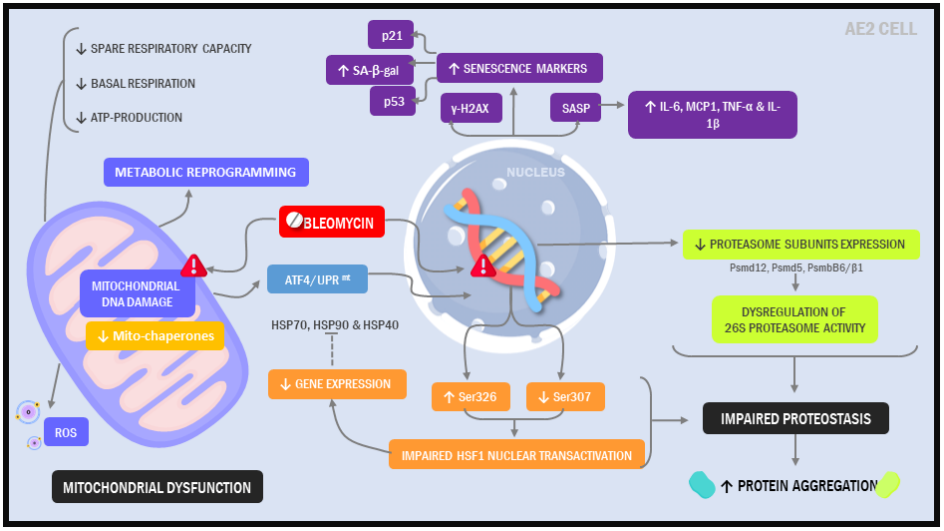
Illustration depicting cellular and molecular changes in MLE12 cells undergoing our 7-day injury model.

Here, our objective was to create a model that more closely resembles the alveolar epithelium in IPF patients. Further, we sought to develop a model that was not just easy to implement but also did not require highly specialized tools or reagents, hoping to facilitate its widespread implementation in the laboratory setting. Using a standard immortalized alveolar epithelial cell line and a simple 2-hit, 7-day injury protocol, we found that phenotypic and functional characteristics of the alveolar epithelium in IPF were readily induced (Figure 8).

Combined, the findings presented in our model recapitulate major pathologic elements described in IPF. Cellular senescence has emerged as a major contributor to the development and progression of IPF [15–17, 28, 29]; in our model, we detected upregulation in multiple cellular senescence markers, including p21, p53, γ-H2AX and SA-βgal. We also observed upregulation in various senescence associated secretory components (SASP), which represent an important source of profibrotic and pro-inflammatory signaling molecules in the IPF lung. The presence of this secretory phenotype has been linked to AE2 cells and lung fibroblasts in IPF.

Growing evidence indicates that aging and cellular senescence are tightly interrelated to mitochondrial dysfunction [13, 30]. Accumulation of dysmorphic/dysfunctional mitochondria have been described in various aging tissues, including AE2 cells in the lungs of IPF patients. In addition, senescent IPF lung fibroblasts show diminished oxidative phosphorylation and increased ROS production, possibly perpetuating the senescence phenotype by promoting ROS-mediated molecular damage. In our study, repeated exposure to low dose bleomycin in MLE12 cells increased ROS levels, suggesting a similar auto-amplifying loop of mitochondrial-derived ROS and senescence. Our results support the free radical theory as a potential explanation for the decline in mitochondrial function [2, 31, 32], although we consider that recently proposed mechanisms, such as decreased PINK1 expression should also be investigated.

Another interesting observation was that mitochondrial dysfunction associated with activation of the UPR^mt^, a cellular stress response pathway that senses and clears damaged proteins [33]. Jiang et al recently reported that ATF4 levels were increased in IPF and that upregulation of ATF4 in the alveolar epithelium is sufficient to enhance murine fibrotic remodeling in the lung [34]. Consistent with this report, we found that ATF4 levels were significantly higher in injured cells and this associated with other markers of dysregulated cytoplasmic and mitochondrial proteostasis [35–37]. For example, we observed downstream activation of critical UPR^mt^ pathways, including the Sirt3/FOXO3a/SOD2 axis, which has been linked to other age-related diseases [38–42]. Altogether, these findings highlight the ability of our model to reproduce findings observed in IPF and undercover new pathways that may contribute to the pathogenesis of disease.

Altered proteostasis in IPF is associated with the accumulation of aggregated proteins, defective autophagy, ER stress, and decline in proteasome activity [43–46]. Supporting the notion that our model mimics pathologic changes in IPF, we showed that aggregated proteins accumulate in MLE12 cells undergoing our protocol. Further, we showed that chymotrypsin-, trypsin- and caspase-like activities were significantly reduced and this associated with decreased levels of several protein subunits of the 26S proteasome. Together, these findings suggest that proteasome dysfunction might contribute to protein aggregation in IPF. Emerging evidence suggests that NRF1 is critically important for regulating proteasome activity in response to protein stress. To date, the role of NRF1 has not investigated in IPF. However, our findings suggest that reduced expression of NRF1 could contribute to loss of proteostasis, providing a potential new direction for the field. Interestingly, Sotzny et al. reported an upregulation of NRF1-dependent proteasome expression upon administration of rotenone, a known inducer of mitochondrial dysfunction, highlighting the possibility that mitochondrial dysfunction and loss of proteostasis are mechanistically linked [47].

Degradation of proteins by the UPS is a multi-step process that begins with the covalent linkage of ubiquitin to a lysine residue on the target protein. Ubiquitination is carried out by three different enzymes, E1, E2, and E3, allowing the development of polyubiquitin chain proteins. Ubiquitination of lysine-48 (K48) residue leads to the formation of K-48 polyubiquitin chains which are considered a major signal for proteasome-mediated degradation. Bearing this in mind, we demonstrated that chronically injured MLE12 cells had higher expression of K48-linked-polyubiquitinated chain proteins and accumulation of intracellular protein aggregates. Thus, our findings suggest that the ubiquitin system is appropriately targeting proteins for degradation but defective activation of the 26S proteasome impedes their degradation. We speculate this may explain the upregulation in urea cycle metabolites since this pathway may be needed to maintain nitrogen balance in these cells. Whether the lung epithelium in IPF manifests similar responses will need to be determined.

Heat shock proteins (HSPs) are cytoprotective chaperones that in conjunction with the proteasome are important to maintain proteostasis [48]. Hsp70 levels are decreased in IPF fibroblasts and Hsp70-knockout mice demonstrate accelerated pulmonary fibrosis after bleomycin administration [48]. In contrast, HSP90 is overexpressed in IPF lung fibroblasts and inhibition of this chaperone attenuated the progression of pulmonary fibrosis. We recently demonstrated that reduction in HSF1 activation plays a central role in reducing chaperone levels in lung fibroblasts [49]. In this study, we show that HSF1 activation, was significantly reduced in injured cells. Reduced expression of HSF1 has been shown to cause senescence by eliciting proteostasis collapse, thereby compromising the ability of cells to perform essential activities. It is now appreciated that increased post-transcriptional modifications, induced via phosphorylation can also contribute to impaired HSF1 activity [50, 51]. In support of this concept, we detected a marked increase in Ser307 phosphorylation in our chronically injured cells.

Bleomycin is a chemotherapeutic agent whose actions have been extensively studied. Although multiple mechanisms are linked to its anti-tumor activities, bleomycin is believed to primarily mediate its effects through chelation of metal ions and the subsequent production of toxic reactive oxygen species. With this in mind, our model appears to support the DNA damage theory of aging, in which functional decline with age is thought to be driven by progressive genomic DNA damage. Given the ability to precisely time the initiation of our insults, our model is ideally suited for uncovering the temporal relationship among DNA damage, mitochondrial dysfunction, metabolic dysregulation and loss of proteostasis.

Although we believe our model has many potential uses, we also appreciate it has limitations. For instance, our model does not factor in the interaction of the lung epithelium with other cells or consider the effects of genetics on cell injury responses. Moreover, our protocol is dependent on bleomycin, rather than employing other insults more relevant to the IPF lung, such as cigarette smoke, silica dust or other environment agents, such as viruses. With that said, the use of bleomycin allows findings to be easily translated to mice, as bleomycin remains the most commonly used insult for modeling pulmonary fibrosis. Finally, we recognize that ML12 cells are an SV40 transformed cell line and that findings from these cells may differ from those with other cell lines or primary cells from the mouse, rat or human lung. To address this, future studies will need to validate the significance of our findings by comparing results to other model systems, such as *ex-vivo* lung slices and primary IPF tissues.

In conclusion, we describe a novel model of the IPF lung epithelium that displays many characteristics of the dysfunctional IPF alveolar epithelium. Given its low cost and ease of implementation, we believe our model is ideally suited for uncovering the molecular underpinnings driving mitochondrial dysfunction, cellular senescence and loss of proteostasis in IPF. Further, we believe our model is also ideally suited for high throughput testing of novel pharmacological compounds directed at the alveolar epithelium. This includes drugs aimed at eliminating dysfunction epithelial cells, such as senolytic medications and agents that aim to restore health to the IPF alveolar epithelium.

## MATERIALS AND METHODS

### Cell culture and reagents

Mouse MLE12 cells were obtained from the American Type Culture Collection (ATCC, Manassas, VA) and cultured according to recommended protocols. To induce cellular injury, cells were exposed to different bleomycin exposures, although ultimately we settled on a two-hit, 7-day injury protocol because of its ability to reliably induce features of senescent cells. In brief, this protocol involved exposing cells to two, 24h exposures of low-dose bleomycin (10 μg/ml) (Enzo life science, BML-AP302-0050, 1mg of Bleomycin is equivalent to 1500 IU) on days 1 and 4, followed by 48 hr post-exposure rest periods. Endpoint analyses were performed immediately after the second 48 hr rest period.

### Mitochondrial ROS assessments

Mitochondrial ROS levels were measured using the Mito-Sox Red dye (Invitrogen, Carlsbad, CA). In brief, MLE12 cells were incubated with dye at a concentration of 5 mM for 10 min at 37° C and fluorescence was measured using a microplate reader.

### Mitochondrial DNA damage assay

Mitochondrial (mt) DNA damage was assessed by qPCR as previously described. In brief, polymerase chain reaction (PCR) was performed to amplify short and long forms of mtDNA and genomic DNA (β-globin). Mitochondrial and genomic DNA were quantified using a microplate reader (PerkinElmer, Waltham, MA.) based on Pico-Green excitation and emission wavelengths of 485 and 530 nm, respectively. Results were normalized by calculating the ratio of small to long mtDNA fragments in each sample. Mitochondrial lesions were determined based on the following equation: D= (1-2 (−Δlong−Δshort) × 10,000 (bp)/size of the long fragment (bp).

### Oxygen consumption measurements

Oxygen consumption rate (OCR) was measured using the Seahorse XFp Bioanalyzer (Seahorse Bioscience, Billerica, MA). In brief, MLE12 cells were seeded at a concentration of 30,000 cells/well on XFp cell plates 24 h prior to the initiation of studies. The concentration of FCCP (also known as trifluoromethoxy carbonylcyanide phenylhydrazone), antimycin A, and rotenone were 0.75 μM, 2μM, and 2μM respectively. Oligomycin was used at a concentration of 4 μM. Pyruvate (1 mM) and glucose (25 mM) served as the substrate for studies. Results were analyzed using wave 2.4 software.

### Senescence-associated beta galactosidase (SA-β-gal) detection

β-galactosidase activity was assessed by performing SA-β-gal staining (Dojindo Molecular Technology, Rockville, MD), according to manufacturer’s instructions. To prevent false positives, staining was performed when cells were at or below 70% confluence.

### Protein aggregation assay

The proteostat aggresome detection kit (Enzo Life Sciences, Farmingdale, NY) was used to quantify protein aggregates and aggresomes. In brief, MLE12 cells were washed with 1X PBS and fixed with 4% paraformaldehyde for 10 min at room temperature (RT) before exposure to permeabilization reagent and stained with ProteosStat dye (1:2000 for 30 m).

### Proteasome activity

Chymotrypsin-, trypsin- and caspase-like activities were assayed at RT using 10 μg of protein in the presence of 100 μM Suc-LLVY-aminoluciferin (Succinyl-leucine-valine-tyrosine-aminoluciferin), Z-LRR-aminoluciferin (Z-leucine-arginine-arginine-aminoluciferin and Z-nLPnLD-aminoluciferin(Z-norleucine-proline-norleucine-aspartate-aminoluciferin), respectively (Promega Corporation, Madison WI). The reaction was monitored by performing fluorimetric measurements every 10 min for 1 h (excitation 350 nm, emission 460 nm) using the Synergy HT Multi-Detection microplate reader. Proteasome activity was determined as the difference between the total activity in the presence or absence of 20 μM MG132 (Millipore-Sigma).

### Western blot analysis

Samples (20 μg) were solubilized and loaded on a 10% Tris-HCl-SDS-polyacrylamide gel and run for 1 h at 100 V before proteins were transferred to a nitrocellulose membrane (ThermoFisher Scientific). Membranes then underwent overnight incubation at 4° C with primary antibodies to p-p53^ser15^, β-actin, Lamin B1(Cell Signaling), γ-H2AX, HSF1, NRF1, FOXO3A, SOD2, SIRT3 (Cell Signaling), ATF4 (Invitrogen), CLPP, LONP1, p-HSF1^ser307^ (Abcam), p-HSF1^ser326^ (Bioss antibodies) or p21 (Millipore), at dilutions of 1:1,000 in blocking buffer with 0.1% Tween-20. This was followed by incubation with secondary antibody which was either a Donkey anti-Rabbit (Li-Cor Biosciences) or a Rabbit anti-mouse (Li-Cor Biosciences) antibody at dilution of 1:8,000. After washing, protein bands were visualized using the Odyssey infrared imaging system (Li-Cor Biosciences).

### RNA isolation and real-time quantitative PCR (qRT-PCR)

Gene transcript levels were quantified by real-time PCR. In brief, total RNA was isolated using RNeasy Mini-Kit (Qiagen), according to the manufacturer’s instructions. A summary of primer sets manufactured by Integrated DNA Technologies can be found in Supplementary Table 1. Relative gene expression levels were quantified with the Livak method (2-ΔΔCT) by normalizing expression of the gene of interest to Hprt, demonstrated to be unaffected by bleomycin, and calculating the relative fold differences between experimental and control samples. Assays were completed in technical triplicate.

### Metabolite extraction

The organic solvent extraction protocol was modified and optimized for 10 cm dishes. Briefly, medium was removed from dishes and cellular metabolism was rapidly halted with 700 ul of chilled mixture of 70% methanol and water containing the internal standards, 25 μM d4-citrate, and d3-Leucine. Plates were immediately placed at -80° C for 10 min to ensure complete metabolic quenching, followed by lysis using a cell scraper. Lysates were vortexed and pelleted (10000 x g for 10 min, 30s mix 30s rest) and pellets were washed twice with a quenching mixture before undergoing additional high speed vortexing. Supernatants were collected and dried under a stream of N2 gas followed by resuspension in 250ul of water containing 0.1% formic acid and subsequent analysis by LC-MS.

### Liquid chromatography-mass spectroscopy

Chromatographic analysis was performed in a Waters Acuity UPLC system (Waters Corp., Milford, MA) using Acuity UPLC HSS T3 1.8μm, 2.1×100mm column with a flow rate of 0.4 and 0.6 ml/min for TCA cycle/glycolytic intermediates and amino acids, respectively. The chromatographic separation was achieved using 9 min gradient elution. For TCA cycle-glycolytic intermediates with solvents of A: 0.2% formic acid water and B: 0.1% formic acid acetonitrile, the initial mobile phase composition was 5% B gradually increasing to 90% B in 2.0min, then held constant at 95% B for 0.5 min, and finally brought back to the initial condition of 5% B in 1 min followed by 2 min re-equilibration. For amino acids and urea cycle metabolites solvents of A included: 0.1% formic acid, 95% water/acetonitrile (pH=5.8) and B: 90% acetonitrile/water, the initial mobile phase composition was 0.1% B, gradually increasing to 40% B in 4.0min, gradually increasing to 99.9% B in 2.0min, and finally brought back to the initial condition of 0.1% B in 1 min followed by 1 min re-equilibration. The injection volume of all samples was 5 μL, but for the glycolytic intermediates, we increased volume to 10 μL.

Mass spectrometry detection was performed using a Xevo Triple Quadrupole MS (Waters Corp) equipped with an electrospray ionization source (ESI) operating simultaneously in positive and negative ionization mode for amino acids and TCA cycle-glycolytic intermediates, respectively. For TCA cycle, and glycolytic intermediates: the desolvation gas flow rate was set to 800 l/h at a temperature of 600° C, the cone gas flow rate was set at 150 l/hand the source temperature at 150° C. For amino acids and urea cycle metabolites: the desolvation gas flow rate was set to 550 l/h at a temperature of 450° C, the cone gas flow rate was set at 150 l/hand the source temperature at 130° C. The capillary voltage was set to 2500 volts for positive ion mode; 1000 volts for negative ion mode; the cone voltage was set depending upon each specific MRM for each metabolite. Data was collected in MRM mode by screening parent and daughter ions simultaneously. MRM transition and parameters for each metabolite are listed in Supplementary Table 2. Except for lactate, which was from 1-0.01mM, ten concentrations of mixed standards were prepared by diluting concentrated stock solutions down to 100-0.001μM for all metabolites. The data were validated by the linearity of the standard curves and recovered internal standards from the biological sample extractions. The MS raw data analysis and the calibration curve for each targeted compounds were calculated in the software TargetLynx (Waters Corp., Milford, MA, USA). The collected data were normalized by the protein mass and internal standard of each sample.

### Statistical analysis

Statistics were performed using GraphPad Prism 8.0 software (GraphPad, San Diego, CA). Two-group comparisons were analyzed by unpaired Student’s t-test, and multiple comparisons were performed using two-way analysis of variance (ANOVA) followed by Sidak post hoc analysis. Statistical significance was achieved when P < 0.05 at 95% confidence interval.

## Supplementary Material

Supplementary Tables
