# Supplementary material for: A model of the aged lung epithelium in idiopathic pulmonary fibrosis
Source: Aging (Albany NY). 2021 Jul 8;13(13):16922–37. doi: 10.18632/aging.203291 (PMC8312437; doi:10.18632/aging.203291)
Supplement: Supplementary Tables [file aging-13-203291-s001.pdf]

SUPPLEMENTARY TABLES

Supplementary Table 1. qPCR primers used in this study.

Mouse gene	Forward	Reverse
**Il-6**	TGAGAAAAGAGTTGTGCAATGG	GGTACTCCAGAAGACCAGAGG
**Mcp1**	CCCAGACAGAAGTCATAGCCAC	TGGTTCTTCCGTTGAGGGAC
**Tnf-α**	ATGAGAAGTTCCCAAATGGC	CTCCACTTGGTGGTTTGCTA
**Il1α**	AGGGAGTCAACTCATTGGCG	TGGCAGAACTGAGTCTTCGT
**Hprt**	AGCAGTACAGCCCCAAAATG	ATCCAACAAAGTCTGGCCTGT
**Hspd1**	TTGCTAATGCTCATCGGAAG	AGCGTGCTTAGAGCTTCTCC
**Hspe1**	GGCCCGAGTTCAGAGTCC	TGTCAAAGAGCGGAAGAAACT
**Trap-1**	AGGACGACTGTTCAGCACG	CCGGGCAACAATGTCCAAAAG
**Psmd10**	GAACTGACCAGGACAGCAGAAC	AGCAGAAGCCGCAATATGAAGA
**Psmd12**	TCACAGACCTGCCAGTCAAG	AGGTTTTAGTCAGCCGAGCA
**Psmd5**	GACCCGGAGTTCCTTCAGAG	CAGAGCCCCCATGACACTTC
**Psmd11**	AGGCAGACAGAAGCATTGAAA	GGTCCAAAATCCCATGAAACT
**Psmb6/B1**	GCAGGCTGGACCCTCAAGAA	CAAGCGGATCACCCCTCCACT

Supplementary Table 2. Optimized MS parameters in ion-mode MS and precursor/product transition of analytes.

Metabolite	Q1	Q3	CV*	CE*	MODE
Glycine	75.80	75.80	25	3	POSITIVE
Alanine	89.70	89.70	25	3	POSITIVE
Serine	105.70	60.00	25	9	POSITIVE
Proline	115.70	70.02	25	14	POSITIVE
Valine	117.70	72.00	25	11	POSITIVE
Threonine	119.70	74.00	26	10	POSITIVE
Leucine	131.80	86.07	25	11	POSITIVE
D3-Leucine	134.80	89.07	25	11	POSITIVE
Asparagine	132.80	73.90	25	14	POSITIVE
Ornithine	133.09	70.01	25	14	POSITIVE
Aspartate	133.75	73.90	25	14	POSITIVE
Glutamine	148.07	84.00	25	10	POSITIVE
Lysine	148.07	84.00	25	10	POSITIVE
Glutamate	148.07	102.60	26	10	POSITIVE
Methionine	150.03	104.00	25	11	POSITIVE
Histidine	155.9	110.1	25	10	POSITIVE
Phenylalanine	165.80	119.90	25	14	POSITIVE
Arginine	175.10	70.01	25	14	POSITIVE
Citrulline	176.16	159.08	25	14	POSITIVE
Tyrosine	181.80	135.90	26	10	POSITIVE
Cystine	241.00	151.70	25	14	POSITIVE
Argininosuccinate	291.14	67.01	25	14	POSITIVE
Citrate	190.989	110.989	6	12	NEGATIVE
D4-Citrate	194.968	113.056	32	10	NEGATIVE
Isocitrate	190.8404	172.90	32	10	NEGATIVE
α-Ketoglutrate	144.9681	100.9651	22	8	NEGATIVE
Succinate	116.9042	72.9483	24	10	NEGATIVE
Fumarate	114.9042	70.938	30	8	NEGATIVE
Malate	132.9042	114.9656	34	12	NEGATIVE
Oxaloacetate	130.9042	86.9425	24	8	NEGATIVE
Pyruvate	86.8404	86.8404	62	3	NEGATIVE
Lactate	88.9042	88.9042	20	4	NEGATIVE
G6P/F6P**	258.8404	96.8550	38	14	NEGATIVE
F1,6BP**	338.8404	96.8550	50	18	NEGATIVE
1,3 BPG**	266.7766	96.8550	36	16	NEGATIVE

*Collision Energy (CE), Cone voltage (CV).
**Glucose 6-phosphate (G6P), Fructose 6-phosphate (F6P),
Fructose 1,6-bisphosphate (F1,6BP),1,3-bisphosphoglycerate (1,3 BPG).
